# International prevalence of adolescent non-suicidal self-injury and deliberate self-harm

**DOI:** 10.1186/1753-2000-6-10

**Published:** 2012-03-30

**Authors:** Jennifer J Muehlenkamp, Laurence Claes, Lindsey Havertape, Paul L Plener

**Affiliations:** 1 Department of Psychology, University of Wisconsin, UW-Eau Claire, 105 Garfield Ave, Eau Claire, WI 54702, USA; 2 Department of Psychology, Katholieke Universiteit Leuven, Tiensestraat 102, 3000 Leuven, Belgium; 3 Department of Child and Adolescent Psychiatry and Psychotherapy, University of Ulm, Steinhoevelstr. 5, 89075 Ulm, Germany

**Keywords:** Non-suicidal self-injury, Deliberate self-harm, Adolescents, Prevalence

## Abstract

**Background:**

The behaviours of non-suicidal self-injury (NSSI) and deliberate self-harm (DSH) are prevalent among adolescents, and an increase of rates in recent years has been postulated. There is a lack of studies to support this postulation, and comparing prevalence across studies and nations is complicated due to substantial differences in the methodology and nomenclature of existing research.

**Methods:**

We conducted a systematic review of current (2005 - 2011) empirical studies reporting on the prevalence of NSSI and DSH in adolescent samples across the globe.

**Results:**

Fifty-two studies fulfilling the inclusion criteria were obtained for analysis. No statistically significant differences were found between NSSI (18.0% SD = 7.3) and DSH (16.1% SD = 11.6) studies. Assessment using single item questions led to lower prevalence rates than assessment with specific behaviour checklists. Mean prevalence rates have not increased in the past five years, suggesting stabilization.

**Conclusion:**

NSSI and DSH have a comparable prevalence in studies with adolescents from different countries. The field would benefit from adopting a common approach to assessment to aide cross-cultural study and comparisons.

## Background

Self-injurious behaviours among adolescents are eliciting increased attention and concern around the world. Research indicates that self-injury tends to first occur during adolescence [1] is associated with a range of psychiatric difficulties [2,3], serves multiple interpersonal and intrapersonal functions [e.g.,[4]] and is significantly associated with increased suicidality [5-7]. Despite a plethora of studies with convenience samples, only recently have more reliable epidemiological studies of prevalence estimates emerged. For example, Klonsky [8] conducted a random-digit dialing survey of adults and estimated that 5.9% of the U.S. population has engaged in non-suicidal self-injury within their lifetime. This rate is only marginally higher from prior epidemiological reports from adult samples in the U.S. (4%) [9]. Within one of the largest epidemiological studies of adolescents to date in the U.S. (n = 61,767), Taliaferro and colleagues [10] report a 12-month prevalence estimate of 7.3% for non-suicidal self-injury. In a comparable epidemiological study of adolescents (age 14 - 17 years) within seven European countries, Madge et al. [11] found an average lifetime prevalence estimate of 17.8% and a 12-month prevalence of 11.5% for deliberate self-harm behaviours (DSH; includes self-damaging acts both with/out suicidal intention); although rates varied across countries. Despite utilizing strong survey methodology each of these studies find different prevalence estimates for the behaviour, preventing the field from drawing conclusions about the true epidemiology of self-injury within adolescents.

The existing data suggest that a significant portion of adolescents are likely to engage in self-injury during their lifetime. Yet, there remain a number of inconsistencies within the literature that need to be addressed in order to have a stronger understanding of the true scope of the problem. Two main obstacles in comparing prevalence estimates from different studies are the different assessment methodologies used (sampling, instruments, and time frames) and different classification systems for self-injury. As noted by experts in the field [12-14] several terms are used to define self-injury. The term deliberate self-harm [11]) is frequently employed as a more encompassing term for self-injurious behaviours both with *and *without suicidal intent that have non-fatal outcomes. This term tends to be used predominantly within European countries and in Australia. In contrast, many studies published by researchers within Canada and the United States have employed the term Non-suicidal self-injury (NSSI; the deliberate, self-inflicted destruction of body tissue without suicidal intent and for purposes not socially sanctioned; [1,15]), which explicitly excludes behaviours engaged in with any level of suicidal intention. These two definitions lead to the use of different assessments and inclusion of specific self-injurious behaviours, which likely contribute to the varying prevalence estimates found. For example, in their review of 128 epidemiological studies of suicidal behaviour in adolescents, Evans and colleagues [16] found that rates of suicidality varied based on the definitions employed (9.7% for suicide attempt vs. 13.2% DSH) and whether questionnaires were anonymous or not. These disparate methodologies and definitions also render cross-country/cross-cultural comparisons of self-injury in adolescents difficult. However, it is important to note that recent attempts have been made to further classify DSH as being "with" and "without" an intent to die (e.g., [14,17]) in order to minimize confusion within the field and promote more accurate comparisons across studies. There is more work to be done along this line to improve international understandings of the scope and characteristics of self-injury in adolescents.

Due to difficulties with agreeing upon a shared definition of self-injury, only a few studies [11,18,19] have been conducted that compare prevalence rates of self-injury between countries using the same assessment tool. Whereas congruent rates of NSSI have been reported in a comparison of adolescents from south Germany and the Midwestern U.S. [19] rates of DSH among adolescents of neighbouring countries (namely Belgium and the Netherlands) have been shown to differ significantly [18]. Recently, the "Saving and Empowering Young Lives in Europe" (SEYLE) study has shown tremendous differences in DSH prevalence rates from participating European countries (also including Israel). Rates of repetitive DSH (5 or more acts) have been shown to be highest in Germany (10.4%) and lowest in Romania (1.9%) [20]. Similar differences in DSH prevalence and associated characteristics were found among the countries participating in the "Child & Adolescent Self-harm in Europe" (CASE) study [11]. Being able to identify differing rates between countries/nations for the same behaviour (e.g., using the same definition or assessment tool) is important to advancing the study of self-injury in adolescents because detecting reliable and valid differences can then lead to investigations of cultural factors that differ between countries to shed light on potential protective and risk factors for the behaviour.

The lack of cross-nation comparisons is a striking deficit in the study of self-injury because it precludes drawing conclusions that could inform international policies and efforts to prevent these behaviours among adolescents. Most salient to this concern, however, may be that the DSM-5 is proposing a non-suicidal self-injury disorder [21] that is largely based on data collected from the U.S. and Canada (because these countries utilize the NSSI definition). This proposal has implications for the psychiatric diagnosis and treatment of adolescents throughout the world yet; the data informing this new diagnosis is limited and drawn predominantly from studies utilizing assessment of NSSI only, which may not have relevance within other countries using DSH definitions, leading to potential cultural bias in the diagnosis. The field's inability to ensure that studies of the prevalence and characteristics of DSH and NSSI are compatible calls into question the potential cultural validity of a NSSI disorder diagnosis.

The purpose of the current study was to attempt to address some of the limitations in the existing literature with regards to the lack of studies comparing the prevalence of NSSI and DSH across countries. We aimed to draw a global perspective by including studies with different terminology (e.g., NSSI, self-injury, DSH, self-harm) and different methodology (sample size, assessment tools). The inclusion of these variables permitted us to examine potential sources of bias/error across studies by comparing average prevalence rates according to definition (NSSI vs. DSH), time frame assessed (i.e., lifetime; 12-month; 6-month), and assessment procedure (i.e., behavioural check-list/questionnaire vs. single-item). A secondary aim of the study was to examine whether, within shared definitions (e.g., NSSI, DSH), the prevalence of self-injury has increased or stabilized since an increase in the phenomenon of self-injury has been frequently mentioned in the literature. Yet, a recent five-year cohort study of adolescents in the U.S. found the prevalence of NSSI to be rather stable [22]. We wanted to extend this study and examine trends across multiple countries to evaluate whether or not rates have stabilized or have continued to increase in recent years.

## Methods

To obtain articles for the current study, we conducted electronic searches within the scholarly database search engines of Medline, PsycInfo, PsycArticles, JSTOR, Academic Search Complete, Social Sciences Citation Index, EBSCO, and PubMed. The search terms: "self-injury, non-suicidal self-injury, NSSI, deliberate self-harm, DSH, self-harm, self-mutilation, parasuicide, prevalence, rates, adolescent, and adolescence" were used to locate articles. We restricted the search to peer reviewed, empirical articles published between January 1, 2005 and December 1, 2011. In a second step, we reviewed the reference lists of identified studies as well as those of recent reviews of self-injury (e.g., [1,23,24] to cross-reference and identify articles for review that did not emerge in our initial database search. Abstracts and methods/results sections of the identified papers were reviewed for inclusion and exclusion criteria. Articles were included if they were written in English, reported empirical data collected from adolescents (age range 11-18 years) within community or school settings, clearly defined their definition of self-injury, at least one focus of the study was on determining the prevalence of self-injury, specified the time frame of their assessment of self-injury behaviour, and clearly identified their method of assessment of self-injury. Studies were excluded if the sample included fewer than 100 participants or included populations with pervasive developmental disorders. Additional exclusion criteria included: inability to determine prevalence estimates within a clear time frame, the definition of self-injury was not clear (could not determine behaviours assessed), the data had been reported in an earlier study of the same dataset, inability to access the full text of the article. Studies reporting prevalence within clinical (inpatient/outpatient/emergency department) studies were also excluded (n = 7) because of the biases inherent in selection of patients and adolescents' access to treatment that could artificially skew results.

## Results

Tables 1 and 2 provide a summary of the data obtained from each study. A common feature across studies of NSSI and DSH is that a majority of studies focus on life-time prevalence estimates. While there was considerable variability across samples, a mean lifetime prevalence of 18.0% (*SD *= 7.3) for NSSI behaviour and 16.1% (*SD *= 11.6) for DSH was observed. The difference in mean prevalence was not statistically significant, *t*(18) = 1.07, *p *> .30, between the two definitional groups. This finding indicates that average rates for NSSI among community samples are comparable to rates of DSH within community samples.

**Table 1:** Prevalence Estimates of NSSI in Adolescents by Year of Publication

Study	N-size	Age Range M (SD)*	Assessment^a^	Lifetime Prevalence %	12-Month Prevalence %	6-Month Prevalence %	Country
Csorba et al. (2005) [25]	470	14-18	Ottawa Self-Injury Inventory	5.5			Hungary

Laye-Gindhu & SchonertReichl (2005) [26]	424	15.34 *(1.06)*	Single Item^b ^- Yes/No	13.2			Canada

Izutsu et al. (2006) [27]	477	14.2	Single Item - Yes/No	8.4			Japan

Muehlenkamp & Gutierrez (2007) [28]	540	15.53 *(1.42)*	SHBQ	23.2			USA

Lloyd-Richardson et al. (2007) [29]	633	15.5 *(1.18)*	FASM	28			USA

Yates et al. (2008) [30]	1,036	Grade 9-12	FASM		37.2		USA

Matsumoto et al. (2008) [31]	1,726	14.5 *(1.4)*	Single Item - Yes/No	9.6			Japan

Hilt et al. (2008) [32]	508	Grade 6-8	Single Item - Yes/No		7.5		USA

Nixon et al (2008) [33]	568	14-21	Single Item - Yes/No	16.9			Canada

Plener et al. (2009) [19]	665	14.8 (0.66)	SHBQ	25.6			Germany

Laukkanen et al. (2009) [34]	4,205	13-18	Single Item - Yes/No	11.5			Finland

Muehlenkamp et al. (2009) [22]	1,393	15.48 *(1.38)*	SHBQ	21.4			USA

Lam et al. (2009) [35]	1,618	13-18	Behavior Check List			16.3	China

Ross et al. (2009) [36]	440	15.39 (1.07)	Single Item - interview	13.9			Canada

Brausch & Gutierrez (2010) [37]	373	15.04 (*1.05)*	SHBQ	21.2			USA

Martin et al. (2010) [38]	1203	15-19yr	Single Item - Yes/No	14.1			Australia

Hasking et al. (2010) [39]	393	13-18	Behavior Check List		33.3		Australia

Claes et al. (2010) [40]	150	15.56 (*2.00)*	Self-Harm Inventory (SHI)	30.7			Belgium

Hankin & Abela (2010) [41]	97	13-16	FASM		18		USA

Hay & Meldrum (2010) [42]	426	15 *(2.8)*	Single Item - 4 point scale	17.7			USA

Prinstein et al. (2010) [43]	377	Grade 6-8	Single Item - 6 point scale		7.4		USA

Baetens et al. (2011a) [44]	1,417	15.13 *(1.76)*	Single Item - Yes/No	13.71			Belgium

Baetens et al. (2011b) [45]	251	16.41 *(1.26)*	Self-Harm Inventory (SHI)	29.9			Belgium

You et al. (2011a) [46]	2,435	14.63 *(1.25)*	12 NSSI behaviors		24.9	13.9	China

Taliaferro et al. (in press) [10]	61,767	Grade 9 & 12	Single Item - Yes/No		7.3		USA

You et al. (2011b) [47]	6,374	14.72 (*1.94)*	5 NSSI Behaviors	15			China

Mohl & Skandsen (2011) [48]	2,864	High school	Single-Item - Yes/No	21.5	16.2		Denmark

Note: * Standard Deviations and mean ages were not always reported within sample descriptions. ^a ^SHBQ = Self-Harm Behavior Questionnaire, FASM = Functional Assessment of Self-Mutilation; ^b ^The most common single item wording was: "*Have you ever intentionally hurt yourself on purpose (e.g, cut, burn) without wanting to die" or "Have you every hurt yourself on purpose without suicidal intent?*"

**Table 2:** Prevalence Estimates of Deliberate Self-Harm in Adolescents by Year of Publication

Study	N- size	Age Range M (SD)*	Assessment^a^	Lifetime Prevalence %	12-Month Prevalence %	6-Month Prevalence %	Country
Young et al. (2006) [49]	1,258	11-19	Single question^b ^Y/N	7.1			UK

Sourander et al. (2006) [50]	738	12-15	Single question Y/N	17.2			Finland

Sidharta & Jena (2006) [51]	1,205	14.73 *(1.44)*	Semistructured interview	18	6.1		India

Patton et al. (2007) [52]	3,332	12-15	Single question Y/N		3.7		US & Australia

Lundh et al. (2007) [53]	123	15	DSHI-9item	41.5			Sweden

Brunner et al. (2007) [54]	5,759	14.9 *(0.73)*	Single question Y/N		14.9		Germany

Portzky et al. (2008) [18]	4,431	15.45 *(0.8)*	Single question Y/N	10.4	7.1		Belgium

Portzky et al. (2008) [18]	4,458	15.5 *(0.6)*	Single question Y/N	4.1	2.6		Netherlands

Bjarehed & Lundh (2008) [55]	202	14.1	DSHI-9item	38.35			Sweden

Morey et al. (2008) [56]	3,881	15-17	Single question Y/N	9.1	5.7		Ireland

Nixon et al. (2008) [34]	568	14-21	Single question Y/N	16.9			Canada

Madge et al. (2008) [11]	30,477	14-17	Single question Y/N	8.8	5.7		EU

Larsson & Sund (2008) [57]	2,464	13.7 *(0.58)*	Single question Y/N	2.9			Norway

Laukkanen et al. (2009) [34]	4,205	13-18	Single question Y/N	21.7	10.2		Finland

van Rijsselberghe et al. (2009) [58]	4,500	16	Single question Y/N	10.4	7		Belgium

Shin et al. (2009) [59]	1,857	13.75 *(1.0)*	Single question Y/N			9.21	Korea

Kvernmo & Rosenvinge (2009) [60]	487	13-16	Single question (YSR)	12.5			Norway

O'Connor et al. (2009) [61]	2,008	15-16	Single question Y/N	13.8	9.8		Scotland

Landstedt & Gillander Gadin (2011) [62]	1,663	17	Two questions Yes/No	17.1			Sweden

Lundh et al. (2011) [63]	992	14.2	DSHI-9item			41.5	Sweden

Tsai et al. (2011) [64]	742	16.8 *(1.2)*	Single question Y/N	11.3			Taiwan

Cerutti et al. (2011) [65]	234	16.47 *(1.7)*	DSHI-17item	42			Italy

Kirchner et al. (2011) [66]	1,171	13.92 *(1.33)*	Single Question (YSR)	11.4			Catalonia

Moran et al. (2011) [67]	1802	15.9 *(0.49)*	5 Y/N Behaviors	8.3	5.1	2.2	Australia

Lucassen et al. (2011) [68]	8,002	Secondary Students	Single question Y/N		20.9		New Zealand

Wan et al. (2011) [69]	17,662	12-24	Behavior Check List		17		China

Note: * Standard Deviations and mean ages were not always reported within sample descriptions. ^a ^SHBQ = Self-Harm Behavior Questionnaire, FASM = Functional Assessment of Self-Mutilation. ^b ^Most common wording of the single question was: "*Have you ever deliberately taken an overdose or tried to harm yourself in some other way?" or, "Have you ever intentionally harmed yourself?"*

Another characteristic that appears salient in the current data is that a majority of studies utilize single item assessments for self-injury, regardless if the definition is NSSI or DSH. The assessment format used appears to contribute to very different estimates of the prevalence of self-injury. Among studies of NSSI, those using a single item (dichotomous Yes/No response) found an average lifetime prevalence of 12.5% (*SD *= 4.5) whereas those using multiple item or behaviour checklists found an average prevalence of 23.6% (*SD *= 8.3), which represents a significantly higher rate relative to single item assessments, *t *(14) = 5.00, *p *< .01. A similar pattern is found within the DSH studies, with single item assessments reporting an average prevalence estimate of 12.2% (*SD *= 5.6) compared to a prevalence of 31.4% (*SD *= 14.9) found for behavioural check-list surveys. The difference in prevalence between these two DSH assessment modalities is statistically significant, *t*(6) = 3.17, *p *< .03, indicating that the type of assessment tools used are contributing potential bias to estimates of self-injury within adolescent populations.

Given the apparent influence of assessment on lifetime prevalence estimates, we re-ran our analyses comparing the mean lifetime prevalence rates between NSSI and DSH by assessment method. The results confirmed that while behaviour based assessments yield higher prevalence estimates than single item assessments, the mean prevalence of NSSI within multi-item assessments (*M *= 23.6; *SD *= 8.3) did not significantly differ from DSH rates (*M *= 31.4; *SD *= 14.9) estimated with multi-item measures, *t *(5) = 1.29, *p *> .25. The same finding emerged when comparing the single item assessment of lifetime NSSI and DSH, *t *(12) = 0.24, *p *> .80.

With regard to the time-frame in which self-injury is assessed, it appears that prevalence estimates again fluctuate and are strongly influenced by the assessment method. The average 12-month prevalence for NSSI was 19.0% (*SD *= 11.9). However, the studies that used self-report inventories where specific behaviours were presented to participants, an average 12-month prevalence of 28.4% (*SD *= 8.6) was reported. This is in sharp contrast to a 12-month NSSI prevalence of 9.6% (SD = 4.40) when a single item assessment was used, *t*(3) = 4.36, *p <*.03. Among the studies examining DSH, an average 12-month prevalence of 9.5% (*SD *= 5.7) is estimated. While our search results only found two studies assessing 12-month prevalence of DSH with a behavioural scale (vs. single item), the prevalence estimates that included multiple-item behaviour based measures yielded a slightly higher average 12-month prevalence of 11.1% (*SD *= 8.4) than the single item assessments mean of 8.5% (SD = 5.3).

**Figure 1:**
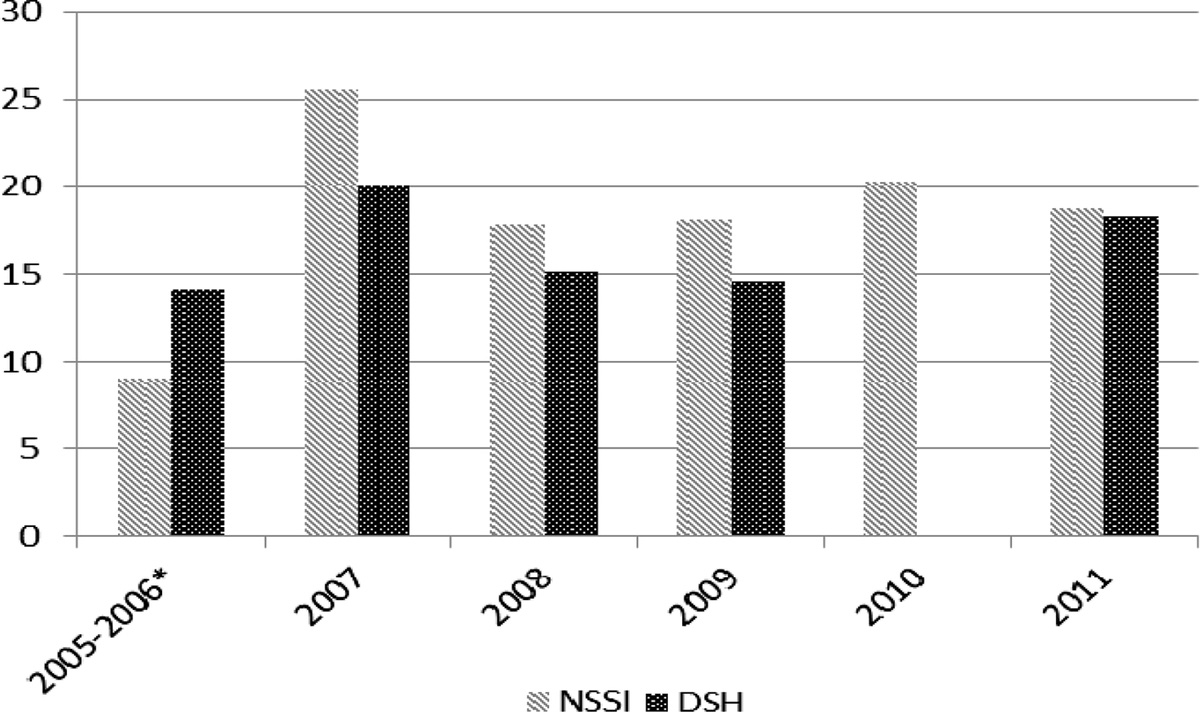
**Average Prevalence of Self-Injury (NSSI and DSH) by Year of Study**. *Note*. NSSI = non-suicidal self-injury; DSH = deliberate self-harm; *Due to limited studies published, years 2005 and 2006 were combined. We were unable to locate any studies of DSH prevalence published in 2010.

To examine whether or not the prevalence of self-injury has been increasing among samples of adolescents, we calculated the average lifetime prevalence rates reported by each study identified, within each year of publication (2005 - 2011), for both NSSI and DSH. Due to limited numbers of prevalence studies, publication years 2005 and 2006 were combined so an average rate could be calculated. Results are presented in Figure 1.

The data appears to support the conclusion that while average prevalence rates are slightly higher among studies published in 2011 compared to 2005, within the past five years the percentage of adolescents reporting NSSI or DSH is relatively consistent and stable. Thus, it appears the global lifetime prevalence of self-injury among community adolescents may have stabilized.

## Discussion

The current study fills a gap in the existing literature by providing an empirical summary of the prevalence of self-injury within adolescent samples across multiple nations and countries. The literature on self-injury has been largely divided into two camps: those who study deliberate self-harm and those who study non-suicidal self-injury. This division has led to difficulties with advancing the field because different definitions and assessment frameworks are used within each nomenclature. The current study helps to bridge the current division by demonstrating that DSH and NSSI have very comparable prevalence estimates within adolescent samples across different countries, which suggests the studies are likely measuring similar phenomena. This finding may also increase comfort with the potential cultural validity of the behavioural criteria being considered by the DSM-5 for a diagnosis of non-suicidal self-injury disorder. Given the many shared features between suicidal self-injurious acts and self-injurious acts without suicidal intent (e.g., [44]) the findings of comparable DSH and NSSI rates may not be surprising, but essential to empirically establish so cross-study comparisons can be made. It remains important to note that essential qualitative and phenomenological differences do distinguish suicidal from non-suicidal self-injurious behaviour [1,6] so continuing to differentiate self-injury with and without suicidal intent is essential to building precise understandings of these behaviours as well as how non-suicidal self-injurious behaviours relate to and influence suicidality.

One of the primary conclusions from our analysis of the prevalence estimates over time is that while at high levels, the percent of adolescents engaging in self-injury appears to have stabilized. Researchers and clinicians have been suggesting that the rates of self-injury are increasing. As the behaviour gained attention both within the clinical literature and media, the production of social/empirical media pertaining to self-injury did significantly increase [70] which could have influenced rates within adolescent populations. Based on the current results, and results reported by Muehlenkamp and colleagues [22] it seems that if actual rates of self-injury had been increasing, they have now largely stabilized in the past five or so years. Some of the variability in prevalence rates observed by individual studies within the literature may partially explain continued exclamations that the behaviour is on the rise in adolescent samples. However, it appears that the variability and perception of increased prevalence is likely the result of assessment bias and sample size.

The current results show that the way in which self-injury is measured has a significant impact on the rates identified. For example, prevalence estimates for NSSI are close to doubled when behavioural check-list measures are used compared to single item questions; and DSH rates using behavioural-based assessment are nearly three-times higher than single item assessments. While it is hard to know if single item assessments are under-estimating the prevalence or if the behavioural assessments are inflating rates, an assessment bias clearly exists. Future work in this area is needed to carefully examine how adolescents understand and interpret the questions being asked of them to ensure they are thinking of potential self-injurious behaviours in the same way experts in this field are. It is well established that how questions are asked of participants has a strong influence on responses. For example, Ross and Heath [71] reported that of the 21.2% adolescents screened for endorsing self-injury, only 13% remained as an adolescent with self-injury after being interviewed about the behaviours. Additionally, data reported by Christl et al. [72] revealed that up to one third of adolescents reporting having attempted suicide at a baseline assessment no longer reported a suicide attempt at the 4-year follow-up. Some adolescents also tend to report alternating between self-injurious behaviour with and without suicidal intent given that suicidal intent is often a transient experience. These results suggest adolescents may be interpreting items assessing NSSI/DSH differently than what the researchers intend and call into question to actual validity of self-report assessments of self-injury within adolescents.

To ensure the validity of our assessments and to avoid unintentionally inflating the estimated scope or severity of self-injury among adolescents, the field of self-injury needs to decide on best-practice assessment processes. Doing so would help to ensure consistency in research, aide with comparisons across studies, reduce artificially elevating concerns about self-injury, and could avoid over-pathologizing adolescents who endorse occasional, mild self-injury. Another option would be for the field to move beyond single-frequency endorsements of self-injury and consider including only those adolescents reporting repetitive acts of a self-injurious behaviour. This approach to assessment and categorization may also help move the field forward in terms of understanding truly pathological self-injury warranting a DSM-5 diagnosis from less pathological self-injury that may represent experimentation and sub-clinical level syndromes. Based on the observations from the current study, we recommend a gold-standard assessment process that would include a single item assessment that if endorsed positively, would be followed up by an interview process to ensure the participant is understanding the behaviour in the same way the field, or researcher, is defining the self-injurious nature of the behaviour.

While the current study fills a void in the self-injury literature, its limitations need to be considered and replication of this study within future years is strongly encouraged. One limitation is that with the proliferation of research on NSSI/DSH it is likely some studies were missed and therefore not included in the current results. Also, we limited studies to those that expressed establishing prevalence as one of the purposes of the study. We recognize most research on self-injury report the percentage of adolescents engaging in the behaviour and thus, not every study conducted on self-injury in adolescents was included. Our decision to exclude studies that did not include assessing prevalence as a study aim may lead to some error in our current results. Another limitation is that we only included studies written in English, so published studies on the prevalence of NSSI/DSH in non-English text journals (e.g., [73]) were not included and does add a layer of cultural bias to our findings. However, we intentionally looked for studies from countries that are less represented in the current self-injury literature to enhance the ability to draw stronger cross-country and global conclusions about the prevalence of self-injury. Still, we recognize that a majority of the studies included in our analysis are from Westernized, developed nations, preventing any analyses between different world regions. Research within non-Western and developing countries are strongly encouraged and greatly needed to obtain a more comprehensive picture of self-injury among adolescents in the world. Lastly, it is important to recognize that there are varying time lags between completion of data collection and actual publication which can extend to some years. Since this study used publication year as the determinant of time, the lag in study publication could skew the observed trends in NSSI/DSH.

The field is currently benefiting from the growth of larger scale, epidemiological surveys of self-injury compared to the convenience samples that typified early prevalence estimates. While the prevalence of adolescents endorsing lifetime rates of self-injury is largely comparable across sample sizes, this growth of large scale studies should contribute to enhancing the field's ability to accurately assess the true prevalence of self-injury as well as improve analysis of trends. Where the field needs to focus efforts is on establishing a consistent standard for assessing self-injury within adolescent samples to ensure construct validity between researchers-adolescent participants. With the increased social and professional attention given to self-injury in recent years the field needs to be cautious not to over-pathologize behaviours. To aide this mission, an agreed upon nomenclature for self-injury (e.g., at least distinguishing self-injury/self-harm with and without suicidal intent) and assessment process that is acceptable across countries is required. At the moment, the current study supports the ability to reliably compare findings of DSH and NSSI with respect to prevalence and trends in the behaviour across adolescent groups and between countries. Given that prevalence seems to have stabilized, the next step is for researchers to start examining specific cultural influences on self-injury so that universal features of the behaviour can be used to inform interventions and culturally-specific variations can inform prevention efforts.

## Conclusion

Comparing rates from community samples reported between 2005 and 2011, the prevalence of NSSI and DSH seems to be very similar, thus adding validity to the proposed DSM 5 diagnosis. Prevalence rates seem to have stabilized within the last years, showing no further increase of NSSI and DSH.

## Competing interests

Jennifer Muehlenkamp, Laurence Claes and Lindsey Havertape declare that they have no competing interests. Paul L. Plener has received travel funding from Lundbeck pharmaceuticals.

## Authors' contributions

All authors were involved in the literature search and in drafting the manuscript. JJM and LH were involved in running the statistical analysis. All authors read and approved the final manuscript.
